# Three-Dimensional Bioprinting for Intervertebral Disc Regeneration

**DOI:** 10.3390/jfb16030105

**Published:** 2025-03-14

**Authors:** Md Amit Hasan Tanvir, Md Abdul Khaleque, Junhee Lee, Jong-Beom Park, Ga-Hyun Kim, Hwan-Hee Lee, Young-Yul Kim

**Affiliations:** 1 Department of Orthopedic Surgery, Daejeon St. Mary’s Hospital, The Catholic University of Korea, Seoul 34943, Republic of Korea; tanvir002@catholic.ac.kr (M.A.H.T.); abdulkhaleque.dream@gmail.com (M.A.K.); rlarkgus21@gmail.com (G.-H.K.); 2 Department of Bionic Machinery, KIMM Institute of AI Robot, Korea Institute of Machinery and Materials, Daejeon 34103, Republic of Korea; meek@kimm.re.kr; 3 Department of Orthopedic Surgery, Uijeongbu Saint Mary’s Hospital, The Catholic University of Korea, Seoul 11765, Republic of Korea; spinepjb@gmail.com

**Keywords:** 3D bioprinting, biomaterials, dECM, IVD

## Abstract

The rising demand for organ transplants and the need for precise tissue models have positioned the in vitro biomanufacturing of tissues and organs as a pivotal area in regenerative treatment. Considerable development has been achieved in growing tissue-engineered intervertebral disc (IVD) scaffolds, designed to meet stringent mechanical and biological compatibility criteria. Among the cutting-edge approaches, 3D bioprinting stands out due to its unparalleled capacity to organize biomaterials, bioactive molecules, and living cells with high precision. Despite these advancements, polymer-based scaffolds still encounter limitations in replicating the extracellular matrix (ECM)-like environment, which is fundamental for optimal cellular activities. To overcome these challenges, integrating polymers with hydrogels has been recommended as a promising solution. This combination enables the advancement of porous scaffolds that nurture cell adhesion, proliferation, as well as differentiation. Additionally, bioinks derived from the decellularized extracellular matrix (dECM) have exhibited potential in replicating biologically relevant microenvironments, enhancing cell viability, differentiation, and motility. Hydrogels, whether derived from natural sources involving collagen and alginate or synthesized chemically, are highly valued for their ECM-like properties and superior biocompatibility. This review will explore recent advancements in techniques and technologies for IVD regeneration. Emphasis will be placed on identifying research gaps and proposing strategies to bridge them, with the goal of accelerating the translation of IVDs into clinical applications.

## 1. Introduction

The intervertebral disc (IVD) plays a decisive part in maintaining the spine’s functionality, serving as a shock absorber to handle axial loads while providing stability and flexibility [1,2,3]. Degeneration of the IVD is an irreversible condition that leads to chronic back pain, a widespread issue that significantly diminishes quality of life and imposes economic strains due to rising healthcare expenses. Current treatments, comprising discectomy, spinal fusion, and artificial disc replacement, focus primarily on symptom management [4,5,6]. However, these procedures often fail to reinstate the natural function and performance of the IVD and can result in complications like reduced mobility and, in severe cases, permanent disability [7,8].

Tissue engineering implies a supporting alternative for curing IVD degeneration. This approach utilizes mesenchymal stem cells (MSCs), osteogenic cells, growth factors, and engineered scaffolds designed to replicate the physical and functional properties of the natural disc [9]. Advances in regenerative remedies and biomaterials have enabled the improvement of IVD scaffolds that mimic the mechanical and biological possessions of native tissues. Furthermore, patient-specific scaffolds are becoming a focal point for improving implant integration and overall treatment outcomes [10].

**Figure 1:**
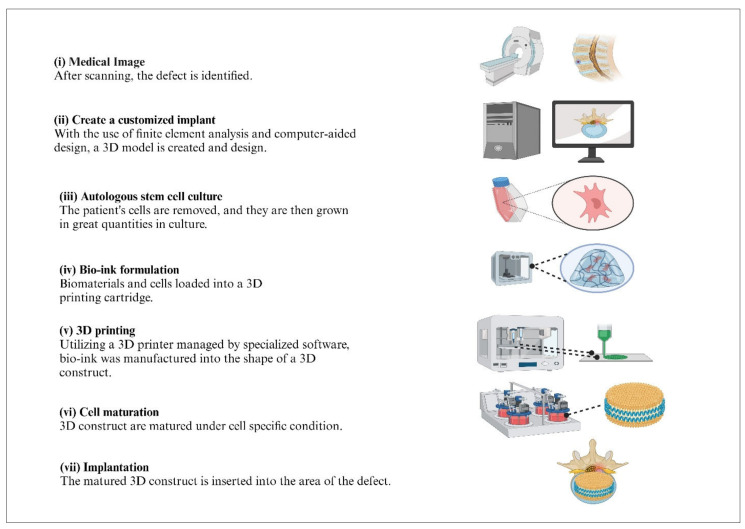
Overview of the 3D bioprinting workflow for regenerative medicine: (**i**) identification of defect via medical imaging, (**ii**) customized implant design using computer-aided tools, (**iii**) expansion of autologous stem cells in culture, (**iv**) formulation of bio-ink with biomaterials and cells, (**v**) fabrication of the 3D construct via bioprinting, (**vi**) cell maturation under specific conditions, and (**vii**) implantation of the matured construct into the defect site.

Among the most exciting technologies in this field is 3D bioprinting, which enables precise placement of biomaterials, bioactive molecules, and living cells [11], as illustrated in Figure 1. This technique employs principles such as biomimicry and mini-tissue assembly to produce constructs that closely resemble the composition and construction of natural tissues [12,13]. For example, researchers have applied fused deposition modelling (FDM) to fabricate customized IVD scaffolds, while other studies have explored the maneuver of polylactic acid (PLA) and acrylonitrile butadiene styrene (ABS) polymers. While these materials provide mechanical support, they fall short in recreating the extracellular matrix (ECM)-like environment necessary for optimal cellular activity [14,15,16]. To address these shortcomings, combining polymers with hydrogels has emerged as a viable strategy. Hydrogels, particularly those derived from natural sources, are highly valued for their ECM-like properties, biocompatibility, as well as competence to uphold cell adhesion and proliferation [1,17].

dECM bioinks are also gaining attention for their ability to replicate the natural microenvironment, enhancing cellular behaviours including differentiation, survival, and migration [18,19]. Notably, injectable materials derived from ECM components have shown promise in promoting the regeneration and repair of spinal discs [20].

Hydrogels are broadly classified into natural and synthetic types. Naturally derived hydrogels, such as those made from collagen, alginate, or gelatin, are favoured for their biocompatibility and biodegradability, though their mechanical properties often require enhancement [21,22,23,24,25,26]. Modifications, such as creating gelatin methacryloyl (GelMA), have addressed these limitations by improving mechanical strength and tailoring biological functionality. Synthetic hydrogels, meanwhile, offer highly customizable properties, but their safety and compatibility require thorough testing [27].

Despite these innovations, achieving an optimal balance of mechanical stability, biocompatibility, and cellular support in IVD scaffolds remains a significant challenge. This review will explore recent advances in 3D bioprinting technologies, bioinks like GelMA and dECM, and other biomaterials to evaluate their potential in IVD regeneration. By identifying gaps in current research, this review aims to propose future directions that could accelerate the clinical application of tissue-engineered IVDs.

## 2. Three-Dimensional Bioprinting

3D bioprinting is a transformative technology that integrates medical science, biology, mechanical engineering, and materials science. It incorporates a wide range of biomedical purposes and, more specifically, the assistance of cell-laden bioinks to fabricate functional tissues. It can be categorized into four key levels: non-biocompatible structures for surgical planning, biocompatible but non-degradable products like prosthetics, biocompatible and degradable items such as biodegradable stents, biomimetic 3D tissues containing living cells for drug testing and the engineering of complex tissues [28,29]. Additive manufacturing of metallic components, ceramics [30], glass [31,32], polymers [33,34], and multimaterials [35] across computer-aided design/computer-aided manufacturing (CAD/CAM) in a layer-by-layer manner dispensing with conventional moulding or machining [36].

Decades ago, pioneers like Vladimir Mironov, Gabor Forgacs, and Thomas Boland proposed merging inventions such as cell patterning with commercial inkjet printing to generate living structures, potentially paving the way for human organ transplantation in the future [37,38].

**Figure 2:**
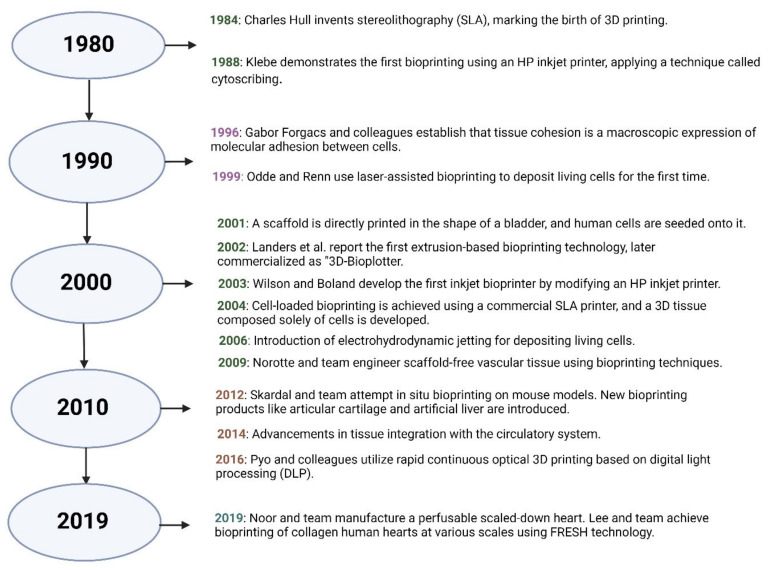
Timeline highlighting major milestones in 3D bioprinting advancements, from the invention of SLA in 1984 to the bioprinting of functional human tissues and organs by 2019.

In 1984, Charles Hull pioneered stereolithography (SLA), enabling the creation of 3D objects directly from digital designs, marking the confinement of 3D printing. By 1988, Klebe had adapted a Hewlett-Packard inkjet printer to deposit cells utilizing a technique known as cytoscribing, showcasing the potential of bioprinting [39]. Significant progress followed in the 1990s. In 1996, Gabor Forgacs and colleagues explored tissue cohesion, identifying it as a macroscopic expression of molecular adhesion between cells [40]. In 1999, Odde and Renn introduced laser-assisted bioprinting to position cells precisely toward constructing intricate structures [41]. By 2001, researchers successfully bioprinted a scaffold with the appearance of a bladder and seeded it with human cells [42]. In 2002, extrusion-based bioprinting was developed by Landers and his team, later commercialized as the 3D-Bioplotter [43]. Between 2003 and 2004, Wilson and Boland modified an inkjet printer to create an early bioprinter, and they also succeeded in using stereolithography to deposit living cells. From 2003 to 2004, Wilson and Boland modified an inkjet printer to create an early bioprinter, and they also succeeded in using stereolithography to deposit living cells [44,45]. The 2000s witnessed further advancements, such as electrohydrodynamic jetting in 2006 for precise cell placement and scaffold-free vascular tissue engineering in 2009 by Norotte and colleagues [46,47]. In 2012, researchers like Skardal achieved in situ bioprinting on animal models, and products such as artificial cartilage and liver tissues emerged [48]. Integration of bioprinted tissues with circulatory systems advanced in 2014 [49]. In 2016, Pyo and colleagues used digital light processing (DLP) for rapid optical bioprinting [50]. By 2019, Noor and his team had bioprinted a functional, scaled-down heart [51]. More recently, Lee and collaborators engineered human hearts using collagen and a hydrogel-based bioprinting approach, demonstrating the potential for scalable, biomimetic organ fabrication [52]. A brief history of 3D bioprinting is shown below (Figure 2).

### 2.1. Three-Dimensional Bioprinting for Tissue Engineering Purposes

Three-dimensional bioprinting using biomaterials is an innovative technology designed to create artificial organs and tissues. Currently, in the research phase, it has been the focus of numerous studies. This technique enables precise control over cell proliferation, attachment, and migration within three-dimensional structures [53]. Various 3D bioprinting techniques are utilized for diverse tissue engineering applications. This study highlights four commonly used methods: SLA and DLP in vat photopolymerization, FFF in material extrusion, SLS in powder bed fusion, and inkjet 3D printing in binder jetting. Table 1 presents the benefits and limitations of different 3D bioprinting techniques used in tissue engineering applications.

### 2.2. Strategies in 3D Bioprinting

Three-dimensional bioprinting relies on three major strategies: biomimicry, autonomous self-assembly, as well as the assistance of mini-tissue building blocks. Each of these is discussed in further depth below.

Biomimicry. Biologically influenced innovation has been developed to solve numerous scientific challenges, such as those in materials science [63], cell culture methods [64], and nanotechnology [64]. With reference to 3D bioprinting, biomimicry is directed to create identical copies of both the cellular and extracellular elements of a tissue or organ [65].

The composite hydrogel demonstrated excellent printability, ensuring strong structural integrity. Muller and his research team developed a composite hydrogel by combining acrylate with unmodified Pluronic F127. This formulation retained the superior printing properties of Pluronic while forming a stable gel through UV crosslinking. As a result, the system enhanced cell viability, increasing it from 62% to 86% by day 14 [66]. Natural polymers such as gelatin and alginate have been integrated into hydrogels to create composites, improving both printability and the cellular viability of the bioprinted constructs [67,68]. Chitosan is widely utilized in 3D printing due to its biocompatibility, biodegradability, and antimicrobial characteristics. However, its use alone is limited by slow gelation and weak mechanical properties. To enhance its performance, gelatin is incorporated into hydrogel composites, promoting improved osteogenic cell proliferation and differentiation. This combination enables excellent printability at room temperature, high structural fidelity in 3D constructs, and superior biocompatibility [69,70]. Polycaprolactone (PCL) is utilized as a reinforcing polymer due to its excellent biocompatibility, relatively long degradation time, and low melting temperature. Its rapid cooling properties help prevent cell damage caused by high-temperature processing during 3D printing. Additionally, graphene/PCL composites have been employed in neural tissue regeneration and have been shown to enhance the chondrogenic differentiation of mesenchymal stem cells (MSCs) in scaffolds fabricated using stereolithography (SL)-based printing techniques [71].

To successfully apply biomimicry in 3D bioprinting, it is vital to replicate the specific functional parts of tissues, including the branching designs of blood vessels and the accurate types and gradients of biomaterials. Achieving this requires microscale replication of biological tissues. A deep comprehension of the microenvironment is vital, which includes the preparation of efficient and supportive cell types, gradients of soluble or insoluble influences, the alignment of the ECM, and the biological forces at play.

Self-assembly (SA). Alternative methods for duplicating biological tissues involve using the advancement of embryonic organs as a model. In the early stages, the cellular components of an emerging tissue generate their individual ECM elements, recruit suitable cell signalling, and undergo autonomous organization and modelling, resulting in the formation of the anticipated biological micro-architecture and function as well [72]. A ‘scaffold-free’ variant of this method utilizes self-assembling cellular spheroids that fuse and organize themselves to replicate emerging tissues. Autonomous self-assembly is a highly intricate process that relies on an in-depth understanding of embryonic tissue development and organogenesis. Additionally, it requires precise environmental control to activate embryonic mechanisms within 3D bioprinted tissues. Okano et al. developed a sheet-based method for cardiac tissue engineering utilizing a self-assembly approach. Following harvest, the layered cardiomyocyte sheets exhibited rapid electrical coupling through functional junction formation. Additionally, after subcutaneous implantation, these pulsatile cardiomyocyte sheets remained viable and continued to develop over an extended period. The self-assembly technique has also been applied to various cell types, including epidermal keratinocytes, kidney epithelial cells, and periodontal ligament cells [73,74,75].

In this autonomous SA process, the cell serves as the major catalyst for histogenesis, guiding the arrangement, placement, and functional and fundamental characteristics of the tissue [76].

**Figure 3:**
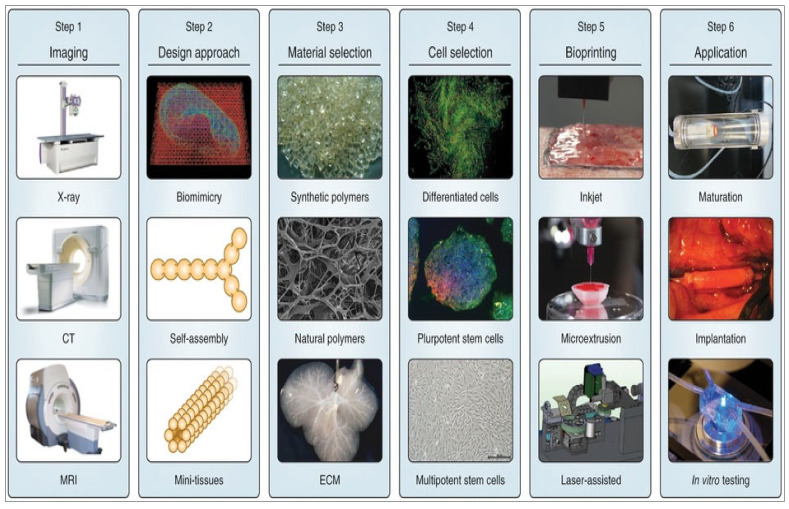
The bioprinting method for creating 3D tissues predictably starts with picturing the injured tissue and environment to direct the design of the printed tissue. Design methodologies such as biomimicry, tissue self-assembly, and MT building blocks are possible to apply individually. Picking the right materials and cell sources is crucial, tailored to the specific form and function of the target tissue. Commonly used materials comprise synthetic or natural polymers and decellularized ECM. These components must work with bioprinting techniques, for example, inkjet, microextrusion, or laser-assisted printing. Certain bioprinted tissues may demand a maturation phase in a bioreactor before they are ready for transplantation, while others can be applied directly for in vitro purposes. Reproduction and adapted with permission from [12] copyright 2014 Nature.

Micro-tissues. This method integrates both biomimicry and self-assembly approaches, making it applicable to both strategies discussed earlier [77]. The idea of mini-tissues (MT) is pertinent to each of the aforementioned tactics for 3D bioprinting, as organs and tissues consist of miniature, functional elements [78]. MT is possibly described as the minutest basic and functional unit of a tissue. These MT were possibly created and combined into an enormous assembly through careful design, as well as SA. There are two primary tactics: the first involves using self-assembling cellular spheres that are organized into a macro-tissue based on biologically intuitive design principles [78]. Secondly, precise, high-resolution replicas of a tissue unit are created and permitted to self-assemble into a functional macro-tissue. An example of this method is the SA of vascular building blocks to construct branched vascular systems [79]. Additionally, 3D bioprinting is employed to precisely replicate efficient tissue parts to develop ‘organs-on-a-chip’. These constructs are sustained and interrelated by a microfluidic network, enabling their practice in drug and vaccine screening or as in vitro models for studying diseases [80,81]. An arrangement of the strategies mentioned above will likely be obliged to bioprint a complex 3D biological construction that incorporates numerous functional, structural, and mechanical elements and properties. Once printed, the construct may be transplanted—sometimes after a phase of in vitro maturation—or it can be set aside for in vitro analysis [12], as illustrated in Figure 3. Gu et al. developed neural tissues by 3D printing human neural stem cells, which subsequently differentiated in situ into functional neurons and supporting neuroglia. A polysaccharide-based bioink composed of alginate, carboxymethyl-chitosan, and agarose was used to encapsulate the stem cells, facilitating their expansion and differentiation. The matured neurons formed synaptic connections and neural networks, exhibiting spontaneous activity. This was accompanied by an increase in calcium response and predominant gamma-aminobutyric acid (GABA) expression.

Additionally, Axel Gunther and his team utilized a microfluidic device to fabricate a resistance artery structure capable of functioning under physiological conditions at 37 °C and a transmural pressure of 45 mm Hg. This device enabled on-chip fixation, long-term culture, and fully automated acquisition of up to ten dose–response sequences from complete mouse mesenteric artery segments, measuring 250 μm in diameter and 1.5 mm in length. When exposed to phenylephrine or acetylcholine, the device demonstrated dose–response relationships that closely resembled conventional myography results [82,83].

### 2.3. Design of 3D Bioprinting Model

Data acquisition for creating 3D models can be achieved through diverse imaging techniques such as X-ray, computed tomography (CT), and magnetic resonance imaging (MRI), or directly through computer-aided design (CAD) software. Once the 3D models have begun, they are sectioned into customizable 2D horizontal slices using specialized software, which then manages the data into particles or filaments based on numerous bioprinting methods. Material selection is crucial, with careful consideration given to biomaterials like cells, growth factors, and hydrogels to design suitable bioinks, which are indispensable for confirming biocompatibility, printability, and mechanical properties. Beforehand to the bioprinting process, it is significant to configure the printing parameters aptly and to monitor the process for necessary modifications.

Over the past ten years, a diversity of bioprinting innovations have been exploited to create tissues and organs by specifically dispensing cells, as well as hydrogels. The following techniques are exemplified based on their underlying mechanisms, with extrusion bioprinting being the most extensively used due to its affordability. A common method within extrusion bioprinting employs multiple nozzles, each designated for dispensing a specific material or cell type. However, this multi-nozzle approach comes with arguments, as switching between materials can prolong printing times and requires precise calibration of each printhead before the printing process starts [84,85]. Moreover, multi-nozzle bioprinting repeatedly directs discontinuities in the extruded filaments, compromising the mechanical integrity of the decisive printed structures. To overcome these issues, recent advancements have concentrated on facilitating the extrusion of several materials using a single nozzle. This method seeks to simplify the printing process, minimize the necessity for frequent recalibrations, and improve the structural consistency and mechanical strength of the printed constructs [84].

Other systems, including inkjet bioprinting, laser bioprinting, and microfluidic-driven bioprinting, are not frequently used because of their comparatively excessive expenses. In this section, we specify a short summary of these technologies, while directing readers to current reviews for an additional designated examination of bioprinting methods [33].

### 2.4. Extrusion Bioprinting

An alternative approach for creating structured 3D hydrogel constructs involves using dispensing systems. This technique typically requires cell-laden hydrogels to be inserted into non-reusable plastic syringes, which are then dispensed onto a building plat form using mechanisms driven by pneumatic pressure, pistons, or screws. Rather than producing discrete droplets, this robotic dispensing approach generates larger hydrogel strands. To maintain the printed structure’s integrity, high-viscosity hydrogels are generally preferred. However, the resolution achievable with robotic dispensing is around 200 μm, which is considerably lower than that achieved by laser- or inkjet-based methods [86].

**Figure 4:**
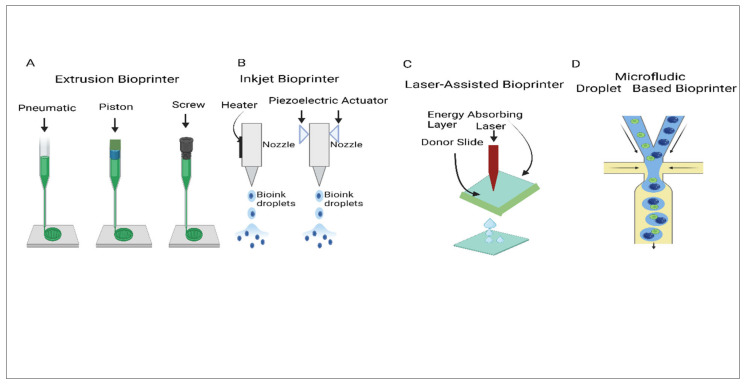
Different schematic diagrams of 3D bioprinting.

Extrusion bioprinting, additionally acknowledged as direct ink writing, is the most prevalent method of 3D bioprinting due to its flexibility and cost-effectiveness. Unlike inkjet printing, which dispenses individual droplets, extrusion bioprinting generates continuous filaments through ongoing extrusion force. This technique accommodates a wide variety of biomaterial viscosities and different cell concentrations, making it appropriate for diverse bioprinting applications [43]. Consequently, researchers favour extrusion-based bioprinting for constructing tissue structures that possess adequate mechanical properties [87,88], as illustrated in Figure 4.

#### 2.4.1. Pneumatic-Driven Extrusion

These techniques engaged compacted air for liquid distributing, typically comprising a syringe filled with bioink joined to an air pump via a connector and pipes. This system is particularly effective with hydrogels exhibiting shear-thinning properties, as they can assert filament consistency after extrusion. In a pneumatic-driven system, maintaining sterility is crucial to prevent contamination of bioprinted constructions. Since the air supplied by the pump may contain impurities or microorganisms, incorporating a high-efficiency filter in the airflow pathway is essential. This filtration system helps remove particulate matter, bacteria, and other contaminants, ensuring a clean and controlled environment for bioprinting. Additionally, using sterile, medical-grade filters can further enhance the reliability of the system, promoting better cell viability and construct integrity. Furthermore, to ensure smooth extrusion, it is critical to add extra liquid or gel-based mediums when dealing with semi-solid or solid-state bioinks to improve their viscosity [89], as illustrated in Figure 4A.

#### 2.4.2. Piston-Driven Extrusion

Mechanical-driven liquid dispensing systems are largely regarded as the most appropriate methods for extruding elevated-viscosity biomaterials, including synthetic and natural high-molecular polymers. Between these systems, extrusion powered by pistons is particularly prevalent, with linked devices readily accessible in the market. In this technique, the piston is linked to a motor via a lead screw, and as the motor operates, rotating motion is converted into linear motion, causing the piston to push bioink out of the nozzle to create filaments [89]. Piston-driven deposition systems offer better control over the hydrogel stream from the nozzle, contrasted to pneumatic systems, as they avoid the variability introduced by the compressed gas volume. To improve the quality of 3D-printed constructs, researchers have investigated deposition methods that utilize highly viscous crosslinking solutions [90], as illustrated in Figure 4A.

### 2.5. Screw-Driven Extrusion

Screw-driven devices characterize an alternative category of mechanical-driven liquid dispensing systems that approach enhanced volumetric control, making them appropriate for extruding biomaterials with higher viscosities. This system functions under a principle related to that of piston-driven devices, but instead of a piston, a screw linked to the motor is exactly utilized for the extrusion process. While screw-driven devices can generate greater pressure, they also pose a risk of harming the cells present in the bioink, necessitating sensible design of the screw components. Furthermore, research has investigated combinations of piston-driven and screw-driven systems; for instance, Visser et al. first printed polycaprolactone (PCL) using a screw-driven approach, followed by the application of a hydrogel on the PCL with a piston-driven method [91].

When contrasted to the pneumatic method, both piston and screw-driven methods offer sophisticated resolution as well as progressed printability for semi-solid or solid-state biomaterials, such as cell aggregates [87], Figure 4A.

### 2.6. Inkjet Bioprinting

An inkjet bioprinter deposits tiny droplets of bioink at specific locations on a substrate [92]. The dual primary techniques exploited for inkjet printing of cells are piezoelectric and thermal inkjet bioprinting [93]. In piezoelectric inkjet printers, piezoelectric crystals generate acoustic waves to push small volumes of liquid through the nozzle [94]. Meanwhile, thermal inkjet systems expel droplets by creating pressure pulses through the vaporization of bioink around a heating element. Research has established that cells are not harmed by the high local temperatures, which can reach up to 300 °C, because of the brief exposure time throughout the printing process, as illustrated in Figure 4B.

### 2.7. Light-Assisted Bioprinting

Laser-assisted bioprinting (LAB) utilizes laser-induced forward transfer (LIFT) principles. Originally conceived for the manipulation of metallic materials, this technology has successfully transitioned to the bioengineering realm, allowing for the precise transfer of biological substances, including peptides, DNA, and living cells [95,96,97]. While not as prevalent as inkjet or micro-extrusion bioprinting, LAB is achieving traction on the grounds of tissue and organ engineering.

The outcome achieved in LAB is affected by several variables. Key issues include laser fluence, which determines the energy imparted to the absorbing layer; surface tension, which influences the behaviour of the biological material; and the wettability of the receiving substrate, which affects how well the materials adhere. Additionally, the space connecting the ribbon and the substrate, known as the air gap, performs a fundamental function, as does the texture and viscosity of the biological layer being transferred [98]. One of the significant advantages of LAB is its nozzle-free design, which effectively eliminates the concern of clogging that can hinder other bioprinting procedures. This characteristic allows LAB to consistently deliver biological materials without interruptions, enhancing the reliability of the printing process. Moreover, LAB demonstrates compatibility with an inclusive scale of viscosities, enabling the precise printing of various biological substances [99,100]. The formulation of individual ribbons for individual cell nature or hydrogel is often a time-intensive process, especially when multiple cell types or materials need to be co-printed. Additionally, the ribbon cell coating method possibly makes precise targeting and positioning of cells difficult, as illustrated in Figure 4C.

### 2.8. Microfluidics Bioprinting

Microfluidics is a well-established tool that began in the 1950s [101] and has been broadly implemented across various fields, where it allows the culture of cells and biomaterials under highly defined and controlled conditions [102]. This has made it feasible to design modern bioprinting systems that provide precise processing and dispensing of low-viscosity materials mixed with cells [103,104].

A benefit of microfluidic bioprinting is its reliance on a well-engrained innovation, which simplifies the improvement of altered print heads. Several commercially accessible bioprinters have now incorporated microfluidic dispensing technology [105], as illustrated in Figure 4D.

### 2.9. Comparison of Different 3D Bioprinting Techniques

Three-dimensional bioprinting enables precise control over cell placement and density, making it well-suited for creating in vitro organ models. Various bioprinting methods have been established for different applications, each with their specific advantages and limitations. Table 2 goes over the advantages and drawbacks of several categories of bioprinting techniques.

## 3. Pathophysiology of Degenerative Intervertebral Disc

The intervertebral disc’s height and internal osmotic pressure fluctuate in response to load-bearing and resting conditions [112], primarily influenced by daily activities and the body’s resting state. These pressure variations drive interstitial fluid movement, facilitating nutrient exchange and metabolism to support intervertebral disc homeostasis [113]. However, disturbances in circadian rhythms can elevate the risk of intervertebral disc degeneration [114]. Most intervertebral disc cells receive nutrients from capillaries in the vertebral body. As disc degeneration progresses, the occlusion of the bone marrow cavity disrupts the connection between the capillaries and the cartilage endplate, leading to its calcification [115]. Degeneration of the intervertebral disc can trigger multiple cellular changes, including alterations in cell type, density, apoptosis, proliferation, senescence, and phenotype [116]. Notochord cells are present during early human development but are gradually replaced by nucleus pulposus (NP) cells as growth progresses. Their disappearance is believed to be linked to the onset of intervertebral disc degeneration [117]. Injecting induced pluripotent stem cells differentiated into notochord cells has been shown to alleviate intervertebral disc degeneration in pig models [118]. The density of nucleus pulposus (NP) cells is lower in degenerated discs compared to healthy disc tissue [119]. Excessive mechanical stress can trigger apoptosis in NP-derived stem cells, leading some researchers to explore anti-apoptotic strategies to slow intervertebral disc degeneration [120]. Additionally, promoting NP cell proliferation has been shown to help prevent disc degeneration [121]. In recent years, advancing research has increasingly focused on the phenotypic traits of NP cells and the connection between cell senescence and intervertebral disc degeneration [122]. With ageing, the likelihood of intervertebral disc degeneration increases, often leading to low back pain as the most common clinical symptom [123]. Research suggests that inflammation is the primary cause of this pain [124]. Recent research indicates that intervertebral disc degeneration is linked to the inhibition of autophagy [125]. Autophagy contributes to preserving the homeostasis of the internal environment [126]. Chen et al. discovered that activating autophagy can reduce cell senescence and apoptosis, ultimately alleviating intervertebral disc degeneration [127]. Subsequent studies have shown that enhancing autophagy can reduce cell senescence and apoptosis, promoting intervertebral disc repair and slowing degeneration [120,128].

## 4. Mechanical Properties of Biomaterials

**Figure 5:**
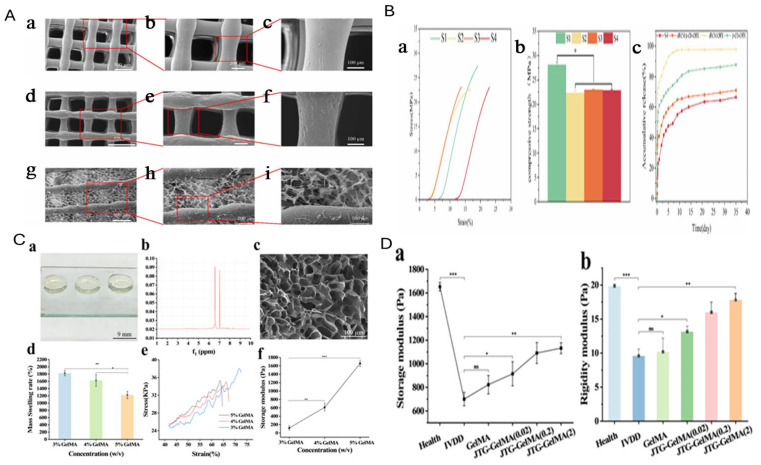
(**A**) SEM images of different scaffold structures at various magnifications, showing S1 (a–c). S2 (d–f). S4 (g–i). (a,d and g) 100×, (b, e and h) 200×, and (c, f and i) 500×, morphology and porosity. Reproduced and adapted with permission from ref [129] under copyright 2012 RSC Advance. (**B**) Mechanical characterization of scaffolds: (a) stress–strain curves, (b) compressive modulus comparison, and (c) time-dependent mechanical behaviour. Reproduced and adapted with permission from ref [129] copyright 2012 RSC Advance. (**C**) Material characterization: (a) macroscopic view, (b) FTIR analysis, (c) porous morphology under SEM, and (d–f) water absorption, swelling behaviour, and degradation profile. Reproduced and adapted with permission from ref [130] copyright 2010 Elsevier. (**D**) Biological evaluation: (a) storage modulus comparison and (b) relative cell viability of different scaffold formulations. Reproduced and adapted with permission from ref [130]. Copyright 2010 Elsevier. (level of significance is * *p* < 0.05, ** *p* < 0.01, and *** *p* < 0.001) (ns: No significance).

Li, S. et al. conducted SEM analysis, revealing that the pores of the PLA scaffold (S1), nHA/PLA scaffold (S2), and nHA/PLA/dECM/b-CD–CHX scaffold (S4) were uniformly sized, with diameters of approximately 250 µm and spacing around 300 µm. As illustrated in Figure 5A-a, the surface of the S1 scaffold appeared smoother compared to S2 and S4. Previous studies have shown that incorporating nano-hydroxyapatite (nHA) into the S2 scaffold resulted in a rougher surface texture (Figure 5A-f). Furthermore, the combination of nHA and composite hydrogels not only maintained the porous nature of dECM hydrogels but also increased the scaffold’s surface area (Figure 5A-i). This enhanced surface area creates a more favourable environment for cell adhesion. When implanted in the jawbone, the composite scaffold supports osteoblast proliferation and facilitates bone regeneration [129].

The mechanical properties of the composite scaffolds in different groups were assessed using compression tests. Figure 5B-a illustrates the stress–strain curves of the S1, S2, S3 (nHA/PLA/dECM scaffold), and S4 scaffolds, from which their compressive strengths were derived (Figure 5B-b). The compressive strengths of S1, S2, S3, and S4 were recorded as (28.14 ± 0.55 MPa), (22.30 ± 0.55 MPa), (22.93 ± 0.14 MPa), and (22.83 ± 0.17 MPa), respectively. The S1 scaffold exhibited significantly higher compressive strength than S2, S3, and S4 (*p* < 0.05), likely due to the reduction in mechanical properties following nHA incorporation. Although S3 and S4 displayed slightly higher compressive strength than S2, the difference was not statistically significant. This slight increase may be attributed to hydrogel filling the scaffold’s pores. However, this variation is not clinically significant, as the composite scaffolds still meet the mechanical requirements for human bone [129].

The GelMA precursor solution was cured by blue light to form a transparent, jelly-like hydrogel with adhesive properties and adjustable shape (Figure 5C-a). The NMR spectrum showed high purity and a 60% crosslinking degree (Figure 5C-b). The hydrogel’s microstructure was uniformly porous (Figure 5C-c), providing ample space for JTG particle adsorption and supporting NP cell growth. The swelling property increased with concentration, but the swelling ratio decreased (Figure 5C-d). Specifically, the 3% GelMA hydrogel swelled significantly more than the 5% version (*p* < 0.01), as higher concentrations formed denser networks with greater crosslinking. Viscoelastic testing showed that 3% GelMA had better elasticity, with a lower storage modulus of 119.78 Pa, while the 5% GelMA had a higher modulus of 1657.04 Pa, indicating increased stiffness (Figure 5C-e) [130].

After 8 weeks of treatment with the JTG-GelMA hydrogel, the mechanical properties of intervertebral disc (IVD) tissue were assessed. The IVDD group showed a significant reduction in the storage modulus and compressive modulus, with a value of only 700.02 Pa. In contrast, the storage modulus of the GelMA group increased to 823.23 Pa. With the addition of JTG, the storage modulus further increased to 1132.32 Pa, showing a significant difference (*p* < 0.01). The compressive modulus also improved (Figure 5D). Compared to the IVDD group, the matrix stiffness of the intervertebral disc gradually increased, suggesting that the JTG-GelMA hydrogel may help restore the mechanical properties of IVD tissue by repairing the nucleus pulposus and promoting ECM synthesis [130].

## 5. Natural Biomaterials

### 5.1. Chitosan

Chitosan (CS) is a biological polysaccharide composed of glucosamine and N-acetylglucosamine, broadly used in damage hemostasis, anti-infection, drug delivery, and gene delivery [131]. These hydrogels are usually softer than native NP tissue [132]. To upgrade the biomechanical properties of CS hydrogels, they are often merged with other biological materials [133]. For instance, a chitosan/dextran hydrogel inserted into the spine has shown no deformation beneath an extensive extent of loads, with Young’s modulus and Poisson’s ratio similar to native IVD underneath unconfined compression [134].

Li et al. designed a thermo-sensitive injectable hydrogel by N-hexanoylating glycol chitosan, which demonstrated a sol–gel transition temperature, influenced by the degree of hexanoylation and polymer concentration. In a porcine model, the hydrogels exhibited no cytotoxicity or negative effects up to four weeks post-implantation, highlighting their promise for intervertebral disc herniation treatment [135]. In a different research, Li, Pinxue, et al. developed a cartilage regeneration methodology applying a hybrid scaffold poised of a CS hydrogel and a 3D-printed PCL structure that incorporates synovium-derived mesenchymal stem cells (SMSCs). This strategy leverages chitosan to direct the recruitment of tetrahedral framework nucleic acid (TFNA), promoting enriched cell proliferation and chondrogenesis. Additionally, this system effectively slows the progression of osteoarthritis following articular cartilage (AC) defects over the long term [136]. Although chitosan-based materials have diverse applications, they are repeatedly combined with other elements to enhance their mechanical strength. CS hydrogels can support cartilage differentiation; however, their limited solubility in a neutral environment and difficulties in processing present significant challenges to their wider use.

### 5.2. Alginate

These hydrogels have been extensively exploited in functions involving drug delivery as well as wound dressing. The stiffness of alginate hydrogels can be modified by adjusting their weight-to-volume (*w*/*v*) concentration. For instance, immersing a 2% (*w*/*v*) alginate solution in CaCl_2_ findings in a hydrogel with mechanical properties alike to those of intuitive IVD tissue [137].

Discectomy may not induce tissue repair, especially in older patients with fewer nucleus pulposus cells (NPCs). A study by Li L et al. in 2020 combined bone-derived mesenchymal stem cells (BMSCs) with ultra-purified alginate (UPAL) gel to enhance intervertebral disc (IVD) regeneration [126]. The approach activated NPCs and BMSCs, promoting differentiation, extracellular matrix production, and preventing degeneration. These findings highlight the potential of BMSCs with alginate-based gel as a regenerative strategy post-discectomy, particularly for elderly patients [138]. In another study by Luo B et al. composite Mel-MBG/SA hydrogel, incorporating mesoporous bioactive glasses and sodium alginate, was developed with enhanced mechanical properties and controlled melatonin release. It mimics the compressive strength of natural intervertebral discs while reducing IL-1β-induced oxidative stress and inflammation. This hydrogel shows potential for intervertebral disc regeneration by combining mechanical support with an anti-inflammatory effect [139].

Crosslinked alginate hydrogels significantly enhance in vivo ECM synthesis while supporting the adhesion, growth, and proliferation of AF, NP, and extended pluripotent stem cell (EP)-derived cells. Among these, EP-derived cells demonstrated the greatest regenerative potential, showing superior NP revival in rabbit models of disc deterioration. Incorporating glucosamine and chondroitin sulphate into alginate hydrogels further promoted NP cell differentiation and matrix assembly [140].

### 5.3. Hyaluronan

Hyaluronic acid (HA), an intuitively appearing polysaccharide in connective tissues, is combined with repeating units of D-glucuronic acid and N-acetyl-D-glucosamine. Its exclusive properties make it vital for various biological processes [141]. Modulating HA concentration has been displayed to improve the stiffness of native NP tissue by maintaining the curved morphology of NP cells. This structural support enhances cell viability and functionality, ultimately promoting extracellular matrix (ECM) production—a critical element in IVD regeneration. Adjusting HA concentration can develop the stiffness of native NP tissue by supporting the curved morphology of NP cells [142,143]. HA-based hydrogels offer considerable clinical potential, particularly when used in conjunction with minimally invasive implantation techniques. Studies have demonstrated that these hydrogels possess anti-inflammatory and matrix-regulating properties, significantly reducing the expression of pro-inflammatory markers in inflamed tissues. Moreover, HA-based treatments upregulate essential ECM elements, while simultaneously downregulating matrix-degrading enzymes like ADAMTS4. These effects create a healthier disc environment, fostering the natural synthesis of IVD matrix components [144].

Research has shown that repair strategies targeting both the annulus fibrosus (AF) and NP—individually or in combination—can effectively address IVD degeneration in ex vivo models, such as rat-tail motion segments after annulotomy and nucleotomy procedures. HA hydrogels can also be merged with other natural materials like gelatin or polyethylene glycol (PEG) to enhance their properties. The addition of gelatin imparts viscoelasticity like native NP tissue without impairing NP cell regeneration. Similarly, combining HA with PEG supports the proliferation of both NP and AF cells, further aiding IVD repair [145,146].

### 5.4. Collagen and Gelatin

**Figure 6:**
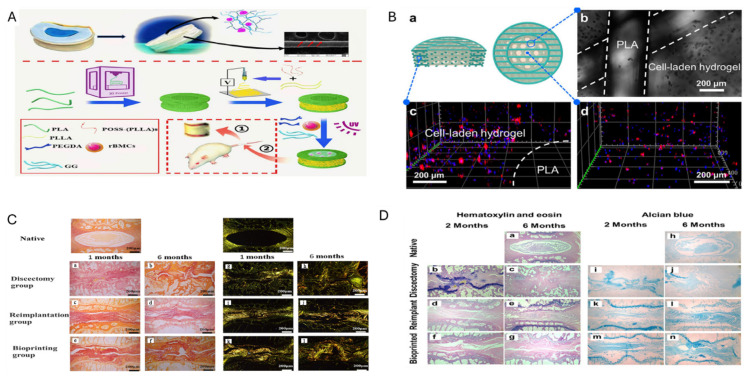
(**A**) A diagrammatic representation of the method involved in initiating a biomimetic artificial IVD) scaffold applying a mixture of 3D printing and electrospinning practices. Reproduced and adapted with permission from [155] copyright 2005 Elsevier. (**B**) Laser Scanning Confocal Microscopy images of the constructs after 14 days of culture: (a) Schematic of the bioprinted IVD construct, showing structural and cell-laden components; (b) High-magnification image displaying the PLA structure and cell-laden hydrogel interface; (c) Confocal image of the cell-laden hydrogel, showing cell distribution and viability; (d) 3D reconstruction of cell-laden hydrogel and PLA scaffold, illustrating cellular organization. Reproduced and adapted with permission from [154] copyright 2012 Elsevier. (**C**) Sirius red staining and Polarized Light Microscopy of disc sections. Native disc tissue at 1 and 6 months, showing intact collagen fibers: (a,b,g,h) Discectomy group, revealing collagen degradation; (c,d,i,j) Reimplantation group, showing partial collagen restoration; (e,f,k,l) The Bioprinting group demonstrates improved collagen alignment. Reproduced and adapted with permission from [155]. (**D**) Hematoxylin & Eosin (H&E) and Alcian Blue Staining: (a) Native disc sections at 2 and 6 months, showing normal tissue structure; (b,c) Discectomy group, displaying tissue degeneration; (d,e) Reimplantation group, indicating partial structural recovery; (f,g) Bioprinting group, showing enhanced disc restoration alcian blue; (h) Native disc section; (i,j) Discectomy group, displaying tissue degeneration; (k,l) Reimplantation group, indicating slightly structural recovery; (m,n) Bioprinting group, demonstrating significant proteoglycan retention. Reproduced and adapted with permission from [154] copyright 2012 Elsevier.

Collagen, another basic ECM element, is found naturally in tissues such as skin, bone, cartilage, blood vessels, teeth, and tendons [147]. Gelatin, derived from collagen through thermal denaturation, shares its biocompatibility and biodegradability, making it commonly used in rehabilitative surgery, drug delivery, and tissue redevelopment applications [148,149]. Collagen I hydrogels exhibit appropriate rheological behaviour, resembling native NP tissue, and their collagen matrix helps repair disc height and mechanical function in the spine [150]. Gelatin, particularly derived from collagen I, is often combined with other components like HA to upgrade its viscoelastic properties [151]. In vivo studies have confirmed that gelatin-based hydrogels are able to prevent the progress of IVD collapse. For example, in NP-suction rabbit models, gelatin hydrogels significantly reduced apoptosis in NP cells compared to untreated IVDs [152,153]. This study developed stem cell-laden, 3D bioprinted scaffolds mimicking native IVD using polylactic acid and a double-network hydrogel, showing excellent mechanical properties, biocompatibility, and regenerative potential in a rat model [154] (Figure 6). In another study, Zhu M et al. exploited a biomimetic artificial IVD scaffold operating 3D printing and electrospinning, combining PLA for the frame, PLLA/POSS fibers for the annulus fibrosus, and GG/PEGDA hydrogel with BMSCs for the nucleus pulposus. The scaffold mimicked the natural IVD’s structure and mechanical properties, with promising results in maintaining disc space and promoting tissue regeneration [155] (Figure 6).

### 5.5. Agarose

Agarose, a polysaccharide, when mixed with water, agarose forms a 3D spiral structure, making it useful in biomedical applications. It has been illustrated to induce none to mild immunological responses in vivo [156].

Agarose has been incorporated into composite hydrogels along with synthetic electrospinning polymers to simulate IVD behaviour in compression and torsion tests. In these composites, agarose mimics the properties of the NP, while the electrospun polymer signifies the AF. Studies using bovine NP cells cultured in agarose hydrogels, infused with transforming growth factor (TGF-β), have exhibited the capability to respond to cyclic compression loads, directing to increased gene expression [157]. While agarose hydrogels only may not be entirely appropriate for IVD regeneration, they are increasingly being integrated into composite scaffolds to recover mechanical properties and provide a more effective NP-AF mimic. As a result, agarose shows promise as a structural component in the advancement of advanced hydrogel scaffolds for IVD regeneration in clinical applications.

## 6. Synthetic Biomaterials

### 6.1. Polyethylene Glycol-Based Hydrogels

Polyethylene glycol (PEG) derivatives represent a crucial class of synthetic hydrogels for NP regeneration. These hydrogels are hydrophilic and biocompatible, exhibiting stealth-like performance in vivo, meaning the immune system is not able to identify them [158,159,160]. While PEG-based hydrogels have biomechanical properties like articular cartilage, including compression, tensile strength, and hydrostatic swelling, non-cell-adhesive properties make them infrequently utilized as standalone materials in regenerative medicine. To address this limitation, PEG hydrogels are often integrated with other synthetic or natural materials [161].

For instance, a multi-component HA-PEG composite hydrogel has been designed to improve matrix synthesis and regulate the activity of NP and AF cells for IVD regeneration. By fine-tuning the molecular weight and hydrogel characteristics, researchers have customized the mechanical properties, metabolite usage, sulfated glycosaminoglycan production, and multi-cell cluster formation within these hydrogels [162].

### 6.2. Polyurethane

PU and derivatives are biodegradable and have been useful in cartilage repair for several years [163]. By adjusting the silk/PU ratio, the degradation rate and mechanical properties of the scaffold can be controlled. In one study, PU/SF scaffolds were injected into the NP cavity to substitute NP tissue in cadaveric porcine spines. SF/PU composite hydrogel as a nucleus pulposus replacement for degenerative disc disease, offering minimally invasive delivery, good mechanical properties, and X-ray/CT/MRI visibility. [164,165].

Li et al. explained a biphasic PU scaffold with rapid swelling properties created utilizing electrospinning techniques. Once implanting this scaffold into a bovine IVD model and subjecting it to dynamic loading for two weeks, the scaffold maintained dynamic compressive stiffness and disc height, showing appropriate stability. The scaffold likewise exhibited promising cytocompatibility for native disc cells. Notably, proteoglycan and type II collagen intensities increased, while type I collagen intensity decreased in the remaining NP tissue, indicating the scaffold’s likely to slow degeneration and uphold the native IVD cell phenotype. These judgments indicate that PU scaffolds could be suitable for nominally invasive methods in promoting IVD regeneration [166].

### 6.3. Poly (-Caprolactone)

Polycaprolactone (PCL), a synthetic polyester permitted by the FDA for practice in humans, exhibits significant potential in various medical applications due to its slow degradation time, ranging from months to years, through the hydrolysis of ester linkages [167,168,169]. PCL scaffolds are promising for accomplished IVD replacement and regeneration since electrospun PCL fibers can mimic the structural affiliation of AF fibers, delivering high mechanical strength [170,171].

**Figure 7:**
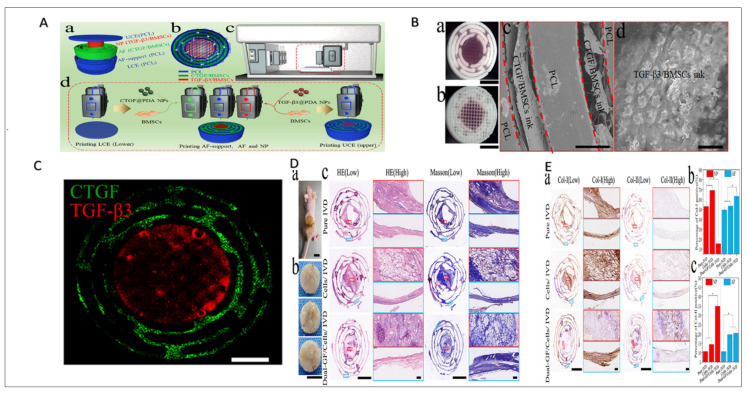
The model was composed of UCE, LCE, NP, AF, and AF-support ((**A**), a), with UCE, LCE, and AF-support sections printed using PCL polymer, 3D bioprinter,and the print process of 3D-bioprinted IVD scaffold ((**A**), b–d). The dual growth factor (GF)–releasing IVD scaffold’s morphology and mechanical properties were analyzed, showing alignment with the designed structure ((**B**), a–d). Fluorescence pictures of TGF-β3 (red) and CTGF (green) in the 3D-bioprinted IVD scaffold (**C**). For in vivo reconstruction, pure IVD, cells/IVD, and dual-GFs/cells/IVD scaffolds were implanted in nude mice, revealing that cells/IVD and dual-GFs/cells/IVD scaffolds improved cartilage, collagen, and chondrocyte formation after 3 months ((**D**), a–c). Notably, the dual-GFs/cells/IVD scaffold in the NP region had higher Col II and lower Col I levels, while the AF region exhibited increased Col I and Col II compared to other scaffolds ((**E**), a–c). Reproduced and adapted with permission from ref [173] copyright 2020 Elsevier.

Research has shown the feasibility of using PCL scaffolds with collagen-hyaluronic acid-based materials to fabricate IVD prostheses with suitable viscoelastic and mechanical properties, while maintaining cell viability [172]. Sun. et al. revealed a model regenerating IVD poses significant challenges due to its intricate structure and function. A 3D-bioprinted scaffold designed for controlled delivery of CTGF and TGF-β3, along with BMSCs, effectively promoted differentiation into nucleus pulposus- and annulus fibrosus-like cells. Both in vitro and in vivo assessments taught encouraging biomechanical and structural results, highlighting its potential for clinical use, with further validation required in large animal studies. (Figure 7) [173].

PCL holds great promise as a component for AF regeneration, particularly because of eminent tensile strength, which reinforces hydrogel scaffolds. This material is especially useful in the fabrication of disc-like structures for whole IVD replacement or regeneration through advanced techniques like 3D printing.

To address these issues, researchers often blend various hydrogels to combine the desirable properties of biodegradability, biocompatibility, processability, and mechanical strength. This approach improves IVD regeneration and tissue repair. High-throughput screening of biomaterials has recognized numerous hydrogel attributes that contribute to their regenerative effects, opening possibilities for designing more effective composite hydrogel scaffolds tailored for clinical treatments [174].

## 7. GelMA for IVD Regeneration

**Figure 8:**
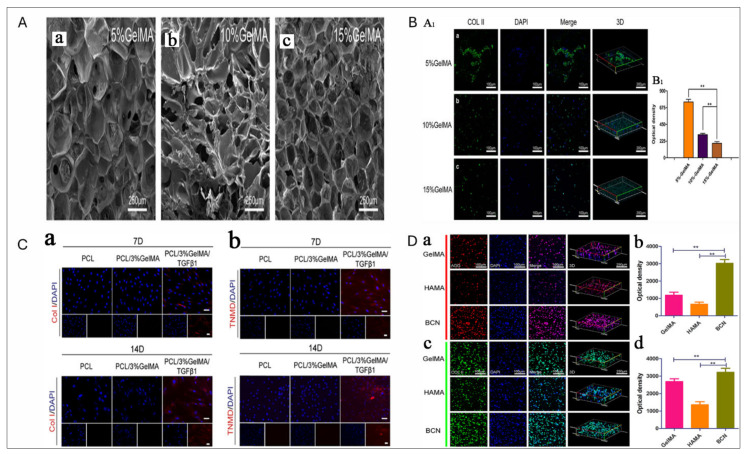
(**A**) SEM images of freeze-dried GelMA hydrogel at three different concentrations: (a) 5% GelMA, showing a porous structure with small, interconnected pores; (b) 10% GelMA, displaying a denser network with reduced pore size; (c) 15% GelMA, revealing a compact structure with minimal porosity. (**B**) Immunofluorescence staining of NPCs in GelMA hydrogels: (A_1_–a) Immunofluorescence staining of NPCs 7 days after encapsulation at 5% concentrations GelMA hydrogels; (A_1_–b) Immunofluorescence staining of NPCs 7 days after encapsulation at 10% concentrations GelMA hydrogels; (A_1_–c) Immunofluorescence staining of NPCs 7 days after encapsulation at 15% concentrations GelMA hydrogels; (B_1_) Quantitative optical density analysis of Col II expression. (** *p* < 0.01). Reproduced and adapted with permission from [177]. (**C**) Immunofluorescence staining for Col I and TNMD expression in TGF-β1-treated groups: (a) Col I/DAPI staining at days 7 and 14 in PCL, PCL/3% GelMA, and PCL/3% GelMA+TGFβ1 groups, showing increased Col I expression in response to TGFβ1; (b) TNMD/DAPI staining at days 7 and 14, indicating enhanced TNMD expression under TGFβ1 treatment. Reproduced and adapted with permission from [178]. (**D**) Morphology and ECM expression of NPMSCs were assessed via aggrecan (a,b) and col-II (c,d) immunofluorescence staining and quantification across three hydrogel types. Reproduced and adapted with permission from [183].

A flawless hydrogel material for IVD repair and regeneration would exhibit the subsequent characteristics: (1) superior biocompatibility and biodegradability; (2) injectability for conservative treatment; (3) in situ remedial to prevent outflow; (4) robust biomechanical properties; (5) effective drug-loading and release capabilities; and (6) stable integration and adherence to the target site to avoid poor localization [175]. GelMA hydrogel, a photosensitive biological hydrogel, possesses favourable biocompatibility, tunable mechanical properties, light-curing properties, and biological activity, making it a popular material in biomedical applications, particularly in orthopedics. GelMA hydrogel facilitates cell and growth factor delivery, supports drug loading, promotes bone fusion, reduces IVD inflammation, and enhances NP tissue regeneration [176]. This provides a biological foundation for the surgical remedy of intervertebral disc degeneration (IVDD). Xu et al. [177] explained using GelMA hydrogels to encapsulate NP cells for improved regeneration. A 5% GelMA concentration provided optimal mechanical properties, supporting cell survival, proliferation, and matrix deposition. These findings highlight the potential of GelMA hydrogels as a controllable scaffold for NP tissue engineering and repairing native NP cells. (Figure 8A-a). Li Z et al. demonstrated a combined strategy using GelMA hydrogel with biomimetic PCL scaffolds loaded with TGF-β1 for AF repair and GelMA with GDF5 for NP restoration, effectively addressing post-discectomy defects. This approach enhanced mechanical strength, induced stem cell differentiation, and restored hydration and extracellular matrix deposition (Figure 8C) [178].

Anita et al. [179] investigated glycosaminoglycan (GAG) deposition and focal adhesion formation of NP cells within hydrogels. Their findings demonstrate that focal adhesion signals play a role in the response of NP cells in hydrogels with integrin binding sites, such as GelMA and type II collagen (Figure 8D).

Mounting evidence suggests that inflammation is more pronounced in IDD than in healthy IVDs, contributing to spinal degeneration progression [180]. GelMA hydrogel can be loaded with anti-inflammatory drugs for targeted delivery to inflamed regions. Liu et al. [181] synthesized a crosslinked ASP-Lip@GelMA hydrogel with anti-inflammatory properties, aimed at inhibiting inflammatory factor expression to restore mechanical stability and prevent IDD recurrence across the entire inflammatory cycle. NP degeneration is a foremost factor in degenerative disc disease (DDD). The elevated water content and fluid pressure attributes of GelMA hydrogels promote oxygen and nutrient infiltration, creating a promising microenvironment for NP cell proliferation and differentiation [182]. Ye Y et al. studies introduced IGF-1 and TGF-β3 genes into NPMSCs using lentivirus, creating dual-gene engineered cells capable of NP differentiation. Delivered via GelMA-HAMA hydrogels, these cells enhanced ECM synthesis and NP differentiation, offering a promising biotherapeutic approach for IVD repair [183].

## 8. Cell Sources for IVD Regeneration

The advancement of functional IVD substitutes relies heavily on selecting optimal cell populations and recreating biomimetic in vitro microenvironments. Contemporary tactics for cell printing involve either placing numerous primary cell categories into specific designs that replicate native tissue or using stem cells to efficiently proliferate and differentiate into the expected cell categories. The printed cells should thoroughly resemble their physiological state in vivo and are imagined to retain their natural functions when placed in optimized environments [184]. Proper regulation of cell proliferation, both in vitro and in vivo, is critical for bioprinting success. If proliferation is too low, the transplanted construct may lose viability, while extreme proliferation can lead to hyperplasia or apoptosis. Controlling proliferation within the construct is fundamental to accomplishing the right balance between functional and supporting cells. Timing is also essential—primarily, a high proliferation rate possibly needed to populate the construct, but over time, it must be regulated to maintain tissue homeostasis without causing hyperplasia. Methods to tackle this involve viral transfection [185] or tiny molecules [186,187] that stimulate cell proliferation and inhibit senescence. In vivo, endogenous stem cells substitute terminally differentiated cells during normal turnover. For bioprinted constructs to function long-term after replacement, they need to maintain cellular homeostasis, self-renew, and respond to tissue damage.

Native cells from degenerated IVDs are not standard owing to their diseased state, though their metabolic activity and proliferation possibly improved using specific growth factors. For example, culturing degenerated human nucleus pulposus cells in alginate with transforming growth factor beta 3 (TGF-β3) and dexamethasone (Dex), or notochordal cell factors, has shown that TGF-β3 and Dex can enrich cell proliferation [188]. These preconditioned cells, largely tested in cast hydrogels, may also be promising for use in 3D bioprinting of NP replacements.

Furthermore, altering NP cells, MSCs are a popular alternative for tissue engineering because of their obtainability and differentiation potential [146]. Nevertheless, the harsh microenvironment of the IVD—marked by high osmolarity, mechanical loads, low oxygen, glucose, acidic pH, and inflammation—negatively impacts MSC viability and functionality, restraining the clinical translatability of MSC-based IVD therapies [146]. Advances in 3D bioprinting may develop MSC survival by incorporating biologically enriched bioinks, which possibly stimulate preconditioning or pre-differentiation of MSCs. Chemical functionalization of bioinks, with growth factors like TGF-β1 or growth differentiation factor-5, can offer additional signals for the embedded cells [188,189]. For instance, Lentivirus-mediated gene transfection using growth factors IGF-1 and TGF-β3 into nucleus pulposus mesenchymal stem cells (NPMSCs) has shown promise for promoting ECM production and delaying IVD degeneration [183,190]. Furthermore, a dual growth factor-releasing scaffold incorporating CTGF and TGF-β3 demonstrated strong compressive strength and supported bone marrow stem cell (BMSC) viability and differentiation [173]. In addition, another study explored BMSC-loaded UPAL gel as a regenerative therapy after partial discectomy in a rabbit model, showing enhanced IVD repair through NPC activation, BMSC differentiation, and ECM production. Both rabbit and human BMSCs demonstrated similar regenerative effects, highlighting the potential for clinical application, particularly in elderly patients with limited endogenous NPCs [138].

More recently, CRISPR/Cas9 genome editing has been used to enhance MSCs’ resistance to harsh environments. By repressing cytokine receptor expression, researchers have minimized the negative effects of inflammation, such as apoptosis and ECM degradation, on MSCs [191]. The potential of CRISPR-modulated MSCs, particularly through multiplexing techniques to target multiple genes simultaneously, is expected to become more prominent in the future [192]. Additionally, research into incorporating extracellular vesicles (EVs), such as those derived from MSCs, into bioinks offers a promising option for growth factor treatments. These EVs could offer an additional capable method to keep cells from apoptosis and inflammation while enhancing ECM production [193].

Pluripotent stem cells (PSCs), whether embryonic or induced (iPSCs), represent a key advancement in tissue regeneration and organ replacement. With their indefinite self-renewal and capacity to differentiate into a large extent of cell types, PSCs have revolutionized regenerative medicine. Embryonic stem cells (ESCs) are able to produce all cell types in the body, though ethical concerns restrict their use in many countries. Induced pluripotent stem cells (iPSCs) offer a less controversial alternative. In 2006, Takahashi and Yamanaka generated the first iPSCs from mouse fibroblasts using transcription factors Oct3/4, Sox2, c-Myc, and Klf4 [194]. They later produced human iPSCs (hiPSCs) using the same approach, which has since been extensively adopted for regenerative therapies [195].

## 9. Categorization and Modelling of Decellularized Extracellular Matrix Scaffold

**Figure 9:**
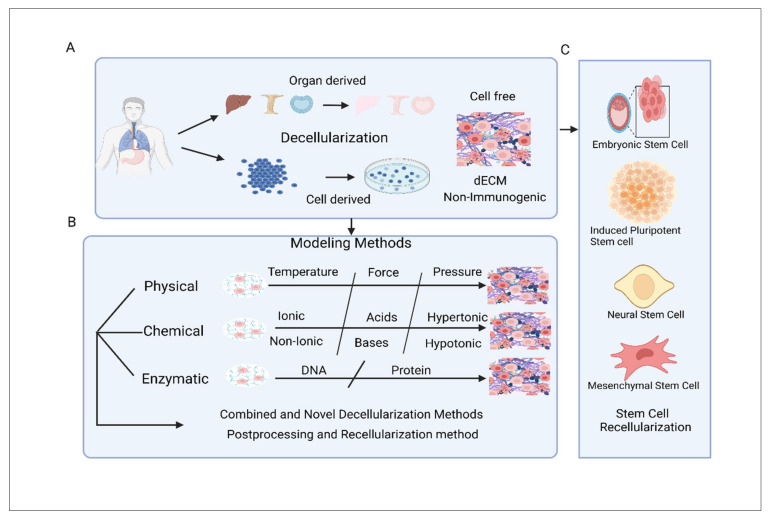
(**A**) Classification of dECM. (**B**) Different fabrication methods for creating dECM from cells and organs. (**C**) Recellularizing dECM scaffolds to create bioengineered grafts for tissue engineering.

In recent tissue engineering strategies, cells are predictably seeded onto pre-formed biomaterial scaffolds, and with the assistance of suitable biochemical and mechanical factors, they generate the desired extracellular matrix (ECM). However, the latest research has increasingly focused on utilizing the tissue microenvironment to guide cell behaviour, aiming to engineer more biomimetic tissues. Instead of applying single-component biomaterial polymers such as hyaluronic acid [196], collagen [197], alginate [197], and polyethylene glycol (PEGs) [198], numerous scholars are now exploring dECM. Decellularized ECM (dECM) scaffolds are categorized as autogenous, allogeneic, or xenogeneic based on their source. Due to tissue limitations and surgical risks, autogenous dECM is less common, while allogeneic and xenogeneic dECM are recurrently used, despite potential issues such as donor site morbidity, structural variations, and immunogenicity from incomplete decellularization. Decellularized ECM (dECM) scaffolds can similarly be separated into two primary classes based on their sources: organ/tissue-derived dECM scaffolds and cell-derived dECM scaffolds (Figure 9A)

### 9.1. dECM Scaffolds Constructed from Organs and Tissues

Organ/tissue-derived decellularized extracellular matrix (O/T-derived dECM) scaffolds are created from precise organs or tissues, retaining their native 3D architecture by removing cellular components that can activate immune responses while preserving the non-immunogenic ECM (Figure 9A). Through a process called decellularization, these tissues are transformed into acellular scaffolds that can be reseeded with appropriate cell types to create tissue-engineered grafts. O/T-derived dECM scaffolds function as reservoirs for localized bioactive molecules and promote crucial cell–matrix interactions, which provide significant improvements for tissue regeneration.

Firstly, native ECM holds bioactive molecules and signalling cues, known as “memory”, factors, which allow O/T-derived dECM to retain tissue-explicit memory. This memory plays a key role in guiding cell behaviour, encouraging cells to undergo tissue-specific differentiation, which is essential for functional tissue regeneration. To illustrate, decellularized heart tissue retains cues that promote cardiomyocyte development, while bone-derived dECM supports osteogenic differentiation [199,200].

Secondly, O/T-derived dECM scaffolds preserve crucial structural components of the ECM, including the architecture of holes and the association of collagen fibres. These structural elements can substantially persuade cellular functions by supporting a microenvironment conducive to cell adhesion, proliferation, and differentiation. The aligned collagen fibres can direct cell migration and upgrade anisotropic tissue formation, while the porous structure allows efficient nutrient and oxygen diffusion, creating optimal conditions for tissue integration and repair. This competence to mimic native tissue environments makes O/T-derived dECM scaffolds a highly promising approach for site-specific tissue regeneration in diverse biomedical applications [201].

### 9.2. dECM Scaffold Constructed from Cell

Cell-derived dECM scaffolds provide an alternative to traditional organ- or tissue-derived dECM scaffolds by offering a tailored cellular niche. When cells are cultured in vitro, they naturally produce their own specific ECM. This ECM can then undergo decellularization to create an acellular matrix, which serves as a substrate for expanding cell populations. This acellular material, referred to as cell-derived dECM, can be recellularized to generate a novel type of decellularized graft [199].

Cell-derived dECM scaffolds have garnered significant attention due to several distinct advantages. Unlike the complex fabrication process associated with organ- or tissue-derived dECM scaffolds, cell-derived dECM scaffolds are comparatively straightforward to produce, exclusively when using progenitor or stem cells as the source. Furthermore, utilizing cell-derived dECM mitigates the risk of pathogen transmission that may occur with allogeneic ECM and minimizes adverse immune reactions commonly associated with xenogeneic ECM [202]. In addition to these benefits, the in vitro production of cell-derived dECM offers flexibility for modifications. This adaptability makes it well suited for integration with other biomaterials, including hydroxyapatite or biphasic calcium phosphate, enhancing its functionality as well as expanding its applications in tissue engineering (Figure 9A). This versatility positions cell-derived dECM as a worthy implement for creating advanced grafts and scaffolds for regenerative medicine [203].

### 9.3. Modelling of dECM Scaffold

Ideal decellularization engages the entire exclusion of all cellular components while protecting the native ECM’s original structure, as well as biochemical and mechanical properties. Currently, no entirely allowed method for decellularization exists, as the process mostly depends on various factors related to the source tissue, including species, age, anatomical location, and tissue size [204,205]. Conversely, scholars have renovated a large diversity of decellularization protocols, including physical, chemical, and enzymatic therapies and a grouping of these methods (Figure 9B).

Physical therapies modulate physical characteristics, such as temperature, force, and pressure, to ease the dipping of detergent solutions, disruption of cell membranes, and elimination of cellular contents. Especially, freeze–thaw cycles, immersion and agitation, and perfusion are extensively applied for decellularization [206,207,208]. Chemical therapies apply detergents and chemical agents to disrupt cellular bonds and eradicate cellular components. Researchers have acquired a wide mixture of protocols, involving Ionic and non-ionic detergents, acids and bases, as well as hypertonic and hypotonic solutions [209,210]. Enzymatic decellularization is exploited to degrade cells and eliminate cellular debris and remnants from the ECM. Considering this, the most frequently applied enzymes are nucleases, deoxyribonuclease, proteases, as well as chelating agents [211].

### 9.4. Combined and Novel Decellularization Methods

The physical, chemical, and enzymatic methods discussed above each offer unique benefits and drawbacks. While all these approaches can effectively remove cellular components, they may also compromise the structure and functional properties of the enduring extracellular matrix (ECM). To minimize damage to the ECM while achieving efficient removal of cellular material, relying on a single method is insufficient. Instead, combining multiple approaches is often necessary to achieve optimal results [212]. For example, combining chemical treatments, such as Triton X-100 and a hypotonic solution, with a physical method like freeze–thaw cycles have been shown to enhance decellularization efficiency. This combined approach resulted in only approximately 1% residual nuclei and 20% residual DNA remaining in the ECM. In contrast, samples that did not include freeze–thaw cycles retained about 20% residual nuclei and 40% residual DNA, highlighting the importance of integrating multiple methods for effective decellularization (Figure 9B) [213].

### 9.5. Postprocessing and Recellularization Method

To improve biocompatibility and remove toxic components from dECM scaffolds, post-processing through sterilization and decontamination is essential. Standard methods of sterilization or disinfection effectively eliminate microorganisms while minimizing toxicity risks [214]. To reinforce mechanical strength and confirm a constant 3D network, decellularized scaffolds typically undergo cross-linking treatments. Although the decellularization process can compromise the biomechanical integrity of the ECM, the utilization of physical and chemical cross-linking techniques helps sustain the 3D structure and improve the mechanical properties of the scaffolds [215]. Additionally, surface modification techniques, including laser treatment, solvent casting combined with particle leaching, and electrospinning, are widely employed to enhance pore density and promote better cell infiltration. These techniques collectively support the functional and structural optimization of dECM scaffolds [216] (Figure 9B).

dECM scaffolds stem cell Recellularization involves the strategic repopulation of acellular ECM scaffolds with precise cell types, particularly stem cells, to create engineered constructs that replicate the structures and functions of natural tissues [217]. Among various options for cell sources, stem cells have earned devotion because of their unique capabilities of differentiation into specific cell lineages and self-renewal through cellular division [218]. Notably, ESCs possess the remarkable ability to maintain pluripotency and differentiate into all three germ layers, which makes them attractive candidates for organ and tissue regeneration [219]. The mixture of ESCs with dECM scaffolds is being extensively investigated across multiple tissue repair contexts, such as those in cardiac, corneal, and renal applications [220,221]. However, ethical concerns and moral dilemmas surrounding the use of ESCs have led to stringent regulations and, in some cases, outright prohibitions on their research and clinical applications.

In response to these challenges, researchers have turned to iPSCs. These cells are reprogrammed from somatic cells using a defined set of transcription factors, enabling them to exhibit gene expression profiles analogous to that of ESCs while possessing an unlimited capacity for proliferation and differentiation [222] (Figure 9C). Recent studies indicate that three-dimensional (3D) dECM scaffolds facilitate improved survival and functional characteristics of cells derived from iPSCs. The relevance of iPSC-derived cells combined with dECM scaffolds is possibly seen across diverse domains of tissue engineering, encompassing organs including the heart, kidney, lung, and pancreas [223,224].

The integration of 3D bioprinting technology has propelled advancements in the field of regenerative medicine [225]. As an economical and hurried scaffold fabrication approach, 3DBP facilitates the capture of tissue macrostructure and acknowledges rigid spatial planning of different biomaterials, bioactive molecules, and cells. As a result, it has been broadly used for developing customized implants for tissue and organ repair [51]. The initial studies, including work by Pati et al. in 2014, marked a pioneering effort in the creation of bioinks from dECM sourced from cartilage, fat, and cardiac tissues, facilitating the production of anatomically relevant shapes and structures [19]. Despite their prospects, dECM-based bioinks face significant limitations, including relatively low mechanical strength and inadequate printability when used at low concentrations, which constrains their effectiveness in biomedical applications [226,227]. To counter these shortcomings, researchers have explored diverse tactics including applying physical support, hybridization with alternative biomaterials, chemical modification, and cross-linking techniques [228]. These methods aim to enhance critical properties including mechanical robustness, structural integrity, viscoelastic behaviour, print fidelity, and overall practicality of dECM-based bioinks [229,230]. For instance, a study developed an injectable decellularized nucleus pulposus (dNP)-based cell delivery system (NPCS) for intervertebral disc (IVD) regeneration, demonstrating biocompatibility, NP-like differentiation of ADSCs, and ECM synthesis. The NPCS retained key matrix components, exhibited native NP-like mechanical properties, and improved disc height and MRI indices in vivo, making it a promising scaffold for NP regeneration [231]. In another study, they developed a decellularized nucleus pulposus matrix/chitosan (DNPM/chitosan) hydrogel crosslinked with genipin for nucleus pulposus tissue engineering. The hydrogel supported nucleus pulposus stem cell (NPSC) growth and incorporated TGF-β3 enhanced collagen-I, collagen-II, and aggrecan expression, making it a promising scaffold for intervertebral disc regeneration [232].

Moreover, the incorporation of active stem cells can further augment the functionality of dECM-based bioinks [226,233,234]. For instance, Jang et al. successfully employed h-dECM (human decellularized extracellular matrix) combined with stem cells in a 3D bioprinting framework. Their results indicated that pre-vascularized cell patches produced via this method provide an optimal environment for in vivo vascularization alongside robust tissue matrix development [235]. Enhancing the printability of dECM inks constitutes a significant advancement within this domain. Studies have demonstrated that adjusting the pH of dECM inks prior to printing and cross-linking can positively impact their mechanical strength and elasticity, thus yielding ECM-derived constructs that are not only structurally sound but also biologically active [227].

## 10. Limitation and Future Perspective

Three-dimensional bioprinting technology has significantly enhanced IVD regeneration in animal models. Scientists have been developing various biomaterials, modifying their chemical structures to minimize potential harm to the body. Despite these advancements, many challenges persist before this technology can be securely applied in human clinical trials. To achieve thriving human applications, biomaterials must be designed with higher precision and biocompatibility to reduce adverse reactions. In addition, Standardized measurements of biomaterials are needed for the successful transplantation of 3D-bioprinted constructs in humans. Several studies explained that different biomaterial ratios can yield outstanding results; however, establishing a standardized measurement is fundamental to guarantee welfare, efficacy, and consistency for clinical purposes.

In IVD regeneration, a crucial challenge is acquiring effective materials for closing the annulus fibrosus. These materials must be not only strong and biocompatible but also capable of preventing the extrusion of the nucleus pulposus. One promising approach concerns using dECM from the patient’s own tissues for 3D bioprinting. This strategy could enhance biocompatibility and ease immune responses. Moreover, the extraction and utilization of mesenchymal stem cells from younger patients could offer meaningful potential, as younger MSCs typically exhibit better proliferation and differentiation competencies. Incorporating growth factors and signalling molecules into these 3D bioprinted structures could further promote cell survival and integration, paving the way for successful IVD regeneration in human trials. For intervertebral disc regeneration, injectable scaffolds are more practical than 3D bioprinted constructs for human application. Unlike 3D bioprinted tissue transplants, which require invasive surgery, injectable scaffolds can be administered directly into the IVD with minimal surgical intervention. This approach not only makes the procedure easier but also reduces recovery time and potential complications associated with traditional transplantation. Additionally, injectable scaffolds can be designed to deliver cells or therapeutic agents completely to the site, promoting successful regeneration within the IVD.

Three-dimensional bioprinting signifies a revolutionary advancement in medical technology; however, it faces numerous significant limitations that must be addressed for effective clinical application. The selection of printable materials remains limited, necessitating the creation of new biocompatible options with specific mechanical and degradation properties. Furthermore, technical arguments such as maintaining cell viability, integrating vascular networks, and ensuring mechanical consistency continue to delay progress. Thus, continuous research and the establishment of clear guidelines are necessary to address these barriers and improve the practical use of bioprinting technologies in healthcare.

To overcome the confines of 3D bioprinting and boost clinical applications, it is crucial to expand the range of biocompatible materials by developing advanced bioinks that mimic natural tissue properties, enhance cell viability, and improve mechanical strength. Optimizing the printing environment and employing advanced techniques like laser-assisted or microfluidic-based bioprinting can enhance cell viability, while strategies such as coaxial printing or incorporating angiogenic factors can help advance functional vascular networks. Improving the mechanical properties of printed constructs with composite materials and using post-printing maturation techniques such as bioreactors can better replicate native tissue environments, thereby advancing the reliability and scalability of 3D bioprinting for medical use.

## 11. Conclusions

In this review, we emphasize diverse forms of 3D bioprinters engaged in tissue engineering, particularly focusing on their applications for IVD regeneration. We also analyze cell sources, biomaterials, decellularized extracellular matrices, and their respective limitations. While 3D bioprinting, biomaterial inks, cells, and decellularized matrices hold great potential, they are still in the early stages of research. Extensive investigation is still required before these technologies are ready for clinical transplantation.

Technological advancements in 3D bioprinting are vital to advance resolution, speed, and compatibility with biologically relevant materials. Instead of adapting preexisting technology, designing specialized 3D bioprinters tailored for individual biological components can expand the range of compatible materials and progress precision in cell and material deposition. Accomplishing clinically relevant tissue sizes also demands faster fabrication. One promising strategy is to create small, functional tissue units that can be scaled up by integrating them within larger, supportive scaffolds.

Commercialization will tend to require scalable, automated robotic systems that streamline the whole bio-fabrication workflow, from printing to assembly [236]. This need extends beyond the printing device itself to encompass the development and synthesis of biomaterials. Special attention should be paid to selecting biomaterials that are biologically compatible with cells and decellularized extracellular matrices, and to identifying materials best suited for specific tissue types to certify successful integration and functionality.

Moreover, new biomaterials that can strengthen vascularization, innervation, and mechanical stability while maintaining biocompatibility are decisive for future clinical applications. By addressing these technological and biological challenges, 3D bioprinting could play a transformative task in regenerative medicine and tissue engineering.

## Figures and Tables

**Table 1:** Outlines the pros and cons of different 3D bioprinting techniques for tissue engineering applications.

Methods	Advantages	Disadvantages	Materials	Ref.
SLA, DLP	Fabricated in both basic and complex designs; High speed with excellent resolution. Does not require support materials.	Costly machinery and materials; Limited to photopolymer materials; Potential cytotoxicity of residual photoinitiators.	PEG, PCL, PEG-co-PDP, PEGDA.	[54,55,56,57]
FFF	Convenient and easy to operate Exhibits strong mechanical properties; No need for solvents.	Restricted to thermoplastic materials; Filament required; Incompatible with living cells.	PCL/PLGA/β-TCP, PCL/PLGA	[58,59]
SLS	No need for support materials; Various biomaterials.	Irregular surface texture; Costly and bulky equipment.	PCL/HA, PCL, HA/PEEK, Titanium	[60,61,62]

**Table 2:** Diverse types of 3D bioprinting techniques with their applications.

Bioprinting Method	Advantages	Limitations	Applications	References
Extrusion-Based Bioprinting	Wide range of biocompatible materials (cell aggregates, hydrogels); Handles viscosities from 30 to 6 × 10^7^ mPa/s; Simple and cost-effective setup.	Lower printing accuracy (~100 μm); Potential for cell damage from shear forces during extrusion.	Creating structures with both high and low cell densities; Research and customized services.	[12,106,107]
Inkjet Bioprinting	Low cost, high precision, and speed; Supports multiple nozzles for simultaneous printing of various cells and materials.	Limited to low-viscosity bioinks, compromising structural integrity; Struggles with high cell density printing, affecting viability and practical application.	Printing different cells and materials simultaneously.	[108].
Laser-Assisted Bioprinting	Nozzle-free, non-contact technique avoiding nozzle clogging and mechanical damage to cells.	High cost and time-consuming.	Printing high-viscosity materials with high cell density and precision.	[99,107,109].
Microfluidic Bioprinting	High precision, and ability to create complex gradients and patterns.	Complexity of microfluidic systems.	Ideal for replicating intricate tissue structures.	[110,111].

## Data Availability

The original contributions presented in this study are included in the article. Further inquiries can be directed to the corresponding authors.
